# Analysis of the Cytoprotective Effect of *Morus alba L*. Fruits as a Means of Transporting the Avulsed Teeth

**DOI:** 10.1155/ijod/6661398

**Published:** 2025-04-22

**Authors:** Mihaela Chirilă, Ioana Suciu, Ecaterina Ionescu, Ionela Daniela Popescu, Eleonora Codorean, Elena Codrici, Lucian Chirilă, Oana Amza, Bogdan Dimitriu, Cornelia Nichita

**Affiliations:** ^1^Department of Endodontics, Faculty of Dental Medicine, University of Medicine and Pharmacy “Carol Davila”, Bucharest, Romania; ^2^Department of Orthodontics and Dento-Facial Orthopedics, Faculty of Dental Medicine, University of Medicine and Pharmacy “Carol Davila”, Bucharest, Romania; ^3^National Institute of Pathology, Victor Babeș, “Victor Babes”, Bucharest, Romania; ^4^Department of Cell Biology and Histology, Carol Davila University of Medicine and Pharmacy, Bucharest, Romania; ^5^Department of Oral-Maxillo-Facial Surgery, Faculty of Dental Medicine, University of Medicine and Pharmacy “Carol Davila” Bucharest, Romania; ^6^National Institute for Chemical—Pharmaceutical Research and Development, Bucharest, Romania

**Keywords:** avulsed teeth, cytoprotective, *Morus alba L*.

## Abstract

This study aims to test the cytoprotective effect of *Morus alba* L. fruit extracts on cell viability and its suitability as a transport medium for avulsed teeth.

**Materials and Methods**. Two *Morus alba* L. fruit extracts were synthesized, E1 and E2. The E1 extract was composed of fresh mulberry fruits, whereas the E2 extract was made from dehydrated fruits. The cytoprotective effect of the E1 and E2 extracts was determined using MTS testing over 5000 and 10,000 cells, after a 48 h incubation period, and sampling times of 1 h and 3 h. The concentrations tested were 50 μg/mL (E1A and E2A), 150 μg/mL (E1B and E2B), and 250 μg/mL (E1C and E2C). The data was analyzed using the one-way analysis of variance (ANOVA) test (*p* < 0.05) and post hoc LSD (least significant difference) analysis (*p* < 0.1).

**Results.** The post hoc LSD analysis based on the concentration of the E1 extract showed statistically differences (*p* < 0.1), and for the E2 extract, the results were highly significant (*p* = 0.011). As a function of concentration between the populations, there are significant differences between E1A and E1B (*p* = 0.071) and between E1B and E1C (*p* = 0.084), and statistically insignificant differences between E1A and E1C (*p* = 0.919). Significant differences were also detected between the E2A and E2B extracts (*p* = 0.047) and between E2B and E2C (*p* = 0.004).

**Conclusions**. Analyzing the preliminary results of our study, we can conclude that the *M. alba* L. extract can be considered a potential transport medium for avulsed teeth.

## 1. Introduction

Avulsion of permanent teeth is an emergency situation in dentistry that presents the most serious consequences in terms of dental trauma. Permanent maxillary central incisors are the most frequently avulsed teeth and the implications are not only functional and esthetic but also psychological [1, 2]. The mechanism of dental avulsion is based on the overload of the periodontal ligament (PDL) that ruptures and can no longer keep the tooth in the alveolus. The actions taken at the place of the accident and promptly following the avulsion determine the prognosis of treatment.

Replanting the tooth in the shortest time possible is often the ideal treatment, but it cannot always be carried out immediately. Adequate management of the avulsion through immediate replantation at the site of the injury and an adequate treatment plan ensures the best chance of survival of the tooth [3]. The therapeutic approach of avulsion of permanent teeth, depending on the stage of root development: with open apex or closed apex, and the extra-alveolar storage period in a dry environment: less than 60 min or more [3].

For the success of replanting, the most important thing is to minimize the extra-alveolar dry time in order to avoid desiccation of the PDL cells that are left on the root surface [4]. This aspect is vital so that osteoclasts do not adhere to the root surface, but instead, it is repopulated with fibroblasts. When approximately half of the PDL has been damaged, complications can occur in the form of root resorption [1]. Additionally, the healing processes after replanting are influenced by age, the degree of root development, and the condition of the pulp. The therapeutic guidelines regarding the treatment of dental avulsion take these aspects into account [1, 3, 4].

Periodontal cells begin to die shortly after the tooth has been aborted, a consequence of exposure to an external, environmental, nonhydrated and therefore dry environment. If the extra-oral time does not exceed 5 min, replantation may have the best prognosis. Periodontal cell death reaches the critical level at about 30 min and increases exponentially after 45–60 min of exposure to a dry environment [3]. The situation improves favorably if, during the extra-oral time interval, until the time of presentation to the doctor and replanting, the tooth is stored in a hydrating storage environment [4].

A storage medium can be defined as a physiological solution that closely replicates the oral environment in terms of osmolarity (230–400 mOsmol/kg) and pH (6.6–7.8), to help maintain the viability of periodontal cells. Among the requirements for an ideal storage environment, very important are: antimicrobial characteristics, maintaining PDL vitality for an acceptable period of time, promoting the clonogenic and mitogenic capacity of periodontal cells, and the absence of antigen–antibody reaction [5–7]. Additionally, a perfect storage environment is defined by several key characteristics such as the ability to help restore the depleted cellular metabolites, a reasonable shelf life, and an affordable price, in addition to the other properties such as biocompatibility and nontoxicity [5].

The storage media used and studied so far can be classified as synthetic (HBSS, ViaSpan, Recitral, physiological serum, etc.) or natural (saliva, milk, water, egg white, propolis, green tea extract, sage extract, mulberry extract, coconut water, aloe vera extract, and pomegranate extract), and so on [5–8]. Studies carried out on the most frequently used storage media have shown that PDL cells maintain their vitality in saliva for a period of 30 min and in milk for 2–3 h. Although providing the necessary hydration, water does not maintain the vitality of PDL cells [9, 10].

The storage medium must meet certain minimum requirements for maintaining the vitality of the PDL cells while still being accessible to a widespread audience. Previous research conducted in this field is usually directed towards identifying and improving natural extracts to obtain efficient and accessible storage media. Plants can be used as a storage medium because of their pharmacological and phytotherapeutic properties to maintain the viability of PDL cells [6].

A study of three Morus species, harvested from Turkey, showed that *Morus alba* L. had the highest total fat content (1.10%) [11]. The main fatty acids present in the berries are linoleic acid (54.2%), palmitic acid (19.8%), and oleic acid (8.41%). The mineral compositions of the samples studied were analyzed using atomic absorbance spectroscopy (AAS) and the *M. alba L*. species showed a 0.75% nitrogen content, 1.668 wt% K, 0.247 wt% P, 0.152 wt% Ca, 0.106 wt% Mg, 0.060 wt% Na, 0.0042 wt% Fe, 0.0038 wt% Mn, 0.0028 wt% Zn, 0.0005 wt% Cu, and a pH of 5.6 [11]. Other studies also found that the juice of the white mulberry fruit is rich in flavonoids, alkaloids, polysaccharides, and antioxidant substances [12].

These results indicate that *M. alba L*. holds significant potential as a storage medium for avulsed teeth, warranting further investigation [11, 12].

The mulberry tree is originally from China and Japan. From Asia, it spread to America and Europe, especially in Southeast Europe. The plant family Moraceae, which contains 24 different species and one subspecies, with at least 100 varieties, also includes the Morus genus to which the mulberry belongs [11]. The best known species are *M. nigra L*., *M. rubra L*., and *M. alba L*.

This study is aimed at analyzing the cytoprotective effect of three white mulberry fruit (*M. alba L*.) extracts. To perform this study, two sample mulberry fruit extracts with geographically different origins (Romania and Turkey) were tested on cell cultures and the results were statistically analyzed, in order to shine light into the cytoprotective characteristics of the fruit extracts and determine the suitability of the *M. alba L*. fruit to act as an effective storage and transport medium for avulsed teeth. The presented investigation was performed under the hypothesis that there is no statistically significant difference between extracts produced from fresh fruits and those produced from dehydrated fruits in terms of cellular activity.

## 2. Materials and Methods

For the present study, fresh fruits of Morus alba L. harvested from Romania, Muntenia region and dehydrated fruits originating from Turkey were used. The fruits of *M. alba L*. are commercial samples, obtained from ecological culture and fresh fruits. The chemical products, reagents for antioxidant activity (AA%)/radical scavenging activity determination, and the equipment used to obtain the extracts from *M. alba L*. fruits are detailed in Table 1.

### 2.1. Preparation of Crude and Selective Extracts From *M. alba L*. (Fruits)

The fruits of *M. alba L*. were air-dried at room temperature, powdered in a mechanical grinder, and extractor with ethanol/water (70:30, v/v). A mass of 100 g of dry fruits was added to 500 mL of solvent and was stirred up for another 45 min, and finally, Soxhlet extraction was carried out. After the extraction procedure was achieved in a Soxhlet installation (three extractive cycles), the vegetal material used was removed, and the combined filtrates (crude extract) were processed by vacuum concentration (Digital Rotary Evaporator RE100-Pro LCD) equipped with a constant temperature water bath until obtaining a concentrated liquid in the ratio of 1:5 (v/v) compared to the initial crude extract (selective extracts).

### 2.2. Total Phenolics Determination

The total phenol content (TPC) of crude ethanol extract and selective extracts was determined using the phenol reagent [11]. Briefly, 50 μL of Folin–Ciocalteu reagent and 500 μL of aqueous sodium carbonate (20%, w/v). The mixture was vortexed and diluted with water to a final volume of 5 mL. After incubation for 30 min at room temperature, the absorbance was measured at 765 nm. The total phenols were expressed as gallic acid equivalents, using a calibration curve of a freshly prepared gallic acid solution.

### 2.3. Total Flavonoids Determination

The total flavonoide content (TFC) of crude ethanol extract and selective extracts was determined spectrophotometrically according to Gundogdu et al. [12] using a method based on the formation of complex flavonoid aluminum, having the maximum absorbance at 415 nm. The same procedure was repeated for the standard solution of rutin and the calibration line was obtained. The flavonoid content was expressed as rutin equivalent using a calibration curve of a freshly prepared rutin solution.

### 2.4. Antioxidant Activities

The AA% was evaluated by chemiluminescence method (CL) using luminol-H_2_O_2_as generator system, in TRIS-HCl, buffer pH = 8.4, using Sirius Luminometer Berhelot–Gmbh Germany. The AA% of samples was calculated using the following relation [13, 14].

(1) $$\begin{array}{c} \text{AA\% }=\frac{I_{0}-I}{I_{0}}100, \end{array}$$

where: *I*_0_ = the maximum CL for standard at *t* = 5 s; *I* = the maximum CL for sample at *t* = 5 s.

There were obtained two extracts noted:

E1-selective extract *M. alba L*. fructus, fruits harvested from fresh local fruits (sampling area Romania—Muntenia region).

E2-*M. alba L*. fructus selective extract, dehydrated fruits (sampling area—ecological culture Turkey).

### 2.5. Testing the Cytoprotective Effect (MTS Test) of *M. Alba L*. Fruit Extracts

The MTS test is a common tool for assessing the modulation of metabolic status in the presence of active compounds, which allows qualitative (effect identification) and quantitative analysis, and is used for preliminary testing of their cytoprotective or cytotoxic effects.

The MTS assay is a colorimetric method based on the use of tetrazole compound, 3-(4,5-Dimethylthiazol-2-yl)-5-(3-carboxymethoxyphenyl)-2-(4-sulfophenyl)-2H-tetrazolium (MTS), which is bioreduced by cells in a colored formazan, soluble in culture medium. This conversion is mediated by NADPH or NADH produced by dehydrogenase enzymes characteristic of metabolically active cells and expresses the oxidative/metabolic status of these cells. Factors that affect the metabolic activity of cells can affect the relationship between cell number and absorption. The assay was performed by using MTS tests on cell cultures of human peripheral blood monocytes (ATCC CRL 9855) and represents a necessary practice to identify and define the safety and efficacy of medicinal plants thresholds for all new potential compounds for consideration in a drug. The analysis is focused on the cytotoxic effects of white mulberry fruit extract *M. alba L*.

## 3. Results

Mulberry fruits possess several potential pharmacological properties, including anticholesterol, antidiabetic, antioxidative, and anti-obesity effects are due to the presence of polyphenol compounds [15]. Many of the bioactivities ascribed to mulberries, such as antioxidant action, hypolipidemic effect, and macrophage activating effect, have also been linked to their phenolic compound composition [16].

The content of polyphenols and flavonoids for the extracts from *M. alba L*. fruits determined in this study is presented in Table 2. The polyphenols related to gallic acid were 4.271 mg/g for the E1 extract and 12.739 mg/g for the E2 extract. Flavonoids reported routinely were 2537 mg/g for E1 extract and 9876 mg/g for E2 extract.

In our study the differences in the determination of total polyphenols, expressed in ac.galic% but also in the determination of flavonoids, expressed in routine% for those E1 and E2 are probably influenced by the high water content (approximately 80%) of the fresh fruits used to obtain the E1 extract and the origin of different geographical areas of the fruit.

The variation of fruit weight, total soluble solids (TSS), pH, and acidity in mulberry fruits could be due to different species, cultivars, rootstocks used, environmental conditions, and the nutritional status of the orchards [10]. The contents of bioactive compounds such as anthocyanins, alkaloids, flavonoids, and polyphenols are dependent on the cultivars [16].

In the present study, several concentrations (C) were tested for the extract E1 produced from fresh fruits and for the extract E2 produced from dehydrated fruits presented in Table 3. The MTS test is used for determining the proliferation potential of in vitro cells. Measurements were made on 5000 and 10,000 cells, with sampling at 1 h and 3 h.

### 3.1. Statistics

The study groups E1 and E2 were compared through one-way analysis of variance (ANOVA) (*p* < 0.05) and post hoc LSD (least significant difference) (*p* < 0.1) was considered statistically significant.

**Figure 1:**
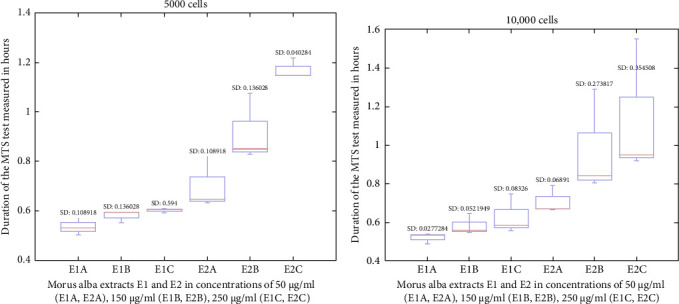
MTS test results read at 1 h: (a) MTS test results on 5000 cells read at 1 h; and (b) MTS test results on 10,000 cells read at 1 h.

The MTS test is used for determining the proliferation potential of in vitro cells. Measurements were made on 5000 and 10,000 cells, with sampling at 1 h and 3 h. During the MTS test at 1 h on 5000 cells, the viability of the cells was maintained with both extracts, with exceptional results for the E2 sample (Figure 1a). At the 10,000 cells test, the result is consistent and shows a characteristic increasing trendline, with a global maximum at the E2C (Figure 1b).

**Figure 2:**
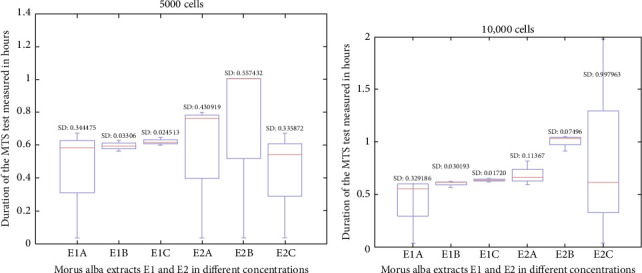
MTS test results read at 3 h: (a) MTS test results on 5000 cells read at 3 h and (b) MTS test results on 10,000 cells read at 3 h.

Testing on 5000 cells at 3 h showed a relatively uniform cellular activity for both extracts, E1 and E2. The maximum efficiency was recorded for the E2B extract (Figure 2a). The degree of absorption of formazan at 3 h on 10,000 cells shows the efficiency of the E1 and E2 extracts, and the maximum effect is manifested by the E2C extract (Figure 2b)

From the statistical analysis (ANOVA), it was proven that there are no significant differences between the 5000 cells and 10,000 cells populations (E1: *p* = 0.645, E2: *p* = 0.979). Depending on the sampling time, at 1 h and 3 h, the differences between the populations are statistically significant for the E1 extract (*p* = 0.023) and statistically insignificant for the E2 extract (*p* = 0.249).

The post hoc LSD analysis based on the concentration of the E1 extract showed statistical differences (*p* = 0.1), and for the E2 extract, the results were highly significant (*p* = 0.011). A significance factor alpha of 0.1 was used in the post hoc analysis.

As a function of concentration between the populations, there are significant differences between E1A and E1B (*p* = 0.071) and between E1B and E1C (*p* = 0.084), and statistically insignificant differences between E1A and E1C (*p* = 0.919). Significant differences were also detected between the E2A and E2B extracts (*p* = 0.047) and between E2B and E2C (*p* = 0.004) according to Table 4.

## 4. Discussion

Violent trauma leading to avulsion causes total or partial damage of PDL, an event that causes inflammation of adjacent tissues, cementum, and alveolar bone. Maintaining as many viable PDL cells as possible reduces postreplantation inflammation in the tissues involved and prevents complications such as stopping root development, ankylosis, inflammatory, or replacement resorption.

Cells can change their functional status in response to stress and maintain their homeostasis. There are two morphological and mechanical models of cell death: necrosis and apoptosis. Apoptosis occurs when the cell dies due to the activation of an internally controlled “suicide” program, which involves an orchestrated disorder of cellular components. If the stress signal is very violent, a particular cell has no choice but to suffer necrotic death, impossible to control genetically or pharmacologically, and rapidly loses cell membrane integrity, releasing the cytoplasmic content and inducing an inflammatory response in the affected tissue. On the other hand, when the stress is moderate, the signal generated will be analyzed by the group of mitochondria, which act as a sensor and have the responsibility to decide whether this cell will continue to live or be eliminated by apoptotic mechanisms, also called postsecondary necrosis [10].

Treatment strategies of avulsion should always be considered in the context of limiting root canal infection and limiting the degree of peri-root inflammation, thus impeding the balance toward a favorable cementum tendency toward healing by bone replacement or inflammatory resorption [17].

Extra-oral time and storage are the critical factors responsible for the prognosis of the avulsed tooth [1, 2, 11, 18]. Both physiological osmolality and pH are important factors in maintaining the viability of PDL cells. It has been reported that cell growth mainly occurs at an osmolality of 230–400 mOsmol/kg and a pH of 6.6–7.8 [6, 12].

In the specialized literature, the characteristics of the *M. alba L*. fruit coming from Turkey had a total fat content of 1.10%. The main fatty acids in mulberry fruits are 54.2% linoleic acid, 19.8% palmitic acid, and 8.41% oleic acid. The mineral compositions of the species were 0.83% nitrogen, 235 mg/100 g phosphorus, 1.141 mg/100 g potassium, 139 mg/100 g calcium, 109 mg/100 g magnesium, 60 mg/100 g sodium, 4.3 mg/100 g iron, 0.4 mg/100 g copper, 4.0 mg/100 g manganese, and 3.1 mg/100 g zinc, and a pH of 5.6 [11].

*M. alba L*. fruits are rich in phenols, flavonoids, anthocyanins, and carotenoids. Phenols possess a broad spectrum of biochemical activities such as antioxidants, antimutagens, and anticarcinogenic properties [19, 20]. Phenols are not present in living plant tissues, but they occur by hydrolysis during fruit processing [12].

Regarding the content of phenolic compounds of *M. alba L*. fruits, gallic acid and routine varied from 0.215 ± 0.001 (mg/g FW) and 1.111 ± 0.002 (mg/g FW) respectively, determined by the spectrometric method [8]. The values for the total phenol of 0.244 – 0.060 were obtained in a study targeting 27 varieties of mulberry [21], compared to 0.4271 for E1, and 1.2739 for E2 in our study. The variation of phenolic compounds in fruits depends on many factors, such as the degree of maturity at harvest, genetic differences, and environmental conditions, during fruit development [11, 12, 20]. It is noteworthy in our study that the extract of fresh native fruits had total polyphenols expressed in gallic acid with 0.0478 higher than the extract of dehydrated fruits.

Ercisli and Orhan found the moisture *M. alba L*. contents was from 71.5%, TSS to 20.4%, and total dry weight to 29.50%. According to the results, *M. alba L*. fruits may be recommended for processing [11]. Yang X, Yang L, and Zheng H reported that the total phenolics, total flavonoids, and anthocyanins in the freeze-dried powder of mulberry fruits were 23.0 mg/g gallic acid equivalents, 3.9 mg/g rutin equivalents, and 0.87 mg/g cyanidin 3-glucoside (C3G) equivalents, respectively. The major flavonol in mulberry fruit powder was rutin (0.43 mg/g), followed by morin (0.16 mg/g), quercetin (0.01 mg/g), and myricetin (0.01 mg/g) [22].

Yang et al. reported that the total phenolics, total flavonoids and anthocyanins in the freeze-dried powder of mulberry fruits were 23.0 mg/g gallic acid equivalents, 3.9 mg/g rutin equivalents, and 0.87 mg/g cyanidin 3-glucoside equivalents, respectively. The major flavonol in mulberry fruit powder was rutin (0.43 mg/g), followed by morin (0.16 mg/g), quercetin (0.01 mg/g), and myricetin (0.01 mg/g). D'Urso et al. identified the phytochemical bioactivity of Morus fruit collected in different areas of the Campania region in Italy, concludes that the phenylpropanoids and flavonols in *M. alba L*. were identified as key antioxidants in their ripe berries [16]. The contents of bioactive compounds such as anthocyanins, alkaloids, flavonoids, and polyphenols are dependent on the cultivars [15].

The amount of polyphenols in *M. alba L*. extracts expressed in mg/100 g differs in Korea, is 257 mg/100 g, in Turkey, 181 mg/100 g, in Pakistan, 1570 mg/100 g [22], and 76.7–180 mg gallic acid/100g in Spain [20].

Regarding the AA%, the extract E2 had the highest values, followed by the extract E1 (Table 1). The concentration of the extracts directly influenced the results for all the extracts. It seems that ethanol extract from *M. alba L*. fruit showed a rapid and concentration-dependent increase in AA% [21]. In similar studies focused on the transport media for avulsed teeth, the typical cell vitality tests used are MTT assay and trypan blue dye exclusion assay [23].

Measurement of the metabolic activity of the cells can be a proof of their viability. The MTS test is based on this principle, and the formazan formed by the activation of mitochondrial dehydrogenase has a high solubility that does not need organic solvents. Therefore, cells in the process of medium elimination do not present a risk of deterioration, so the MTS test has a high level of sensitivity characterized by visibility [23, 24].

*M. alba L*. extracts offered a good cellular cytoprotection in MTS testing at 1 h and 3 h. The differences were statistically significant for the E1 extract for the 1 h measurements (*p* = 0.023). Similar results were recorded by other authors as well, in studies referring to transport media for avulsed teeth [25, 26].

The white mulberry extracts showed that they offer a good cellular cytoprotection in the determinations by the MTS test at 1 h and 3 h. The differences were statistically significant for the E1 extract for the determinations at 1 h compared to those at 3 h (*p* = 0.023). Similar results were reported by other authors in studies on natural transport media for avulsed teeth [25, 26].

The role of the avulsed tooth repair after replantation will depend on the potential repair of each cellular component of the tissues involved, the procedure made the replantation, and specific individual factors. The cytoprotection demonstrated by the mulberry extracts studied here is an important characteristic, which confirms their suitability as a transport medium for avulsed teeth.

Further investigations into the activity of other polyphenols, such as resveratrol, identified in *M. alba L*. fruits and having a strong anti-inflammatory effect are needed. Resveratrol appears to inhibit PDL production of interleukin-8, which plays a central role in initiating and maintaining the inflammatory response [25].

## 5. Conclusions

Analyzing the results of our study, which is a preliminary study, we can conclude that the white mulberry fruit extract can be considered a potential transport medium for avulsed teeth.

The dehydrated *M. alba* L. extract, with a concentration of 250 µg/mL (E2C), showed the best cytoprotection, determined through MTS testing at 3 h. Obviously, additional tests and studies are needed to say with certainty that the extract from *M. alba* L. fruit is a transport medium for avulsed teeth.

Because there is currently no product capable of preserving the vitality of PDL cells with high availability and low cost, specialists must continue and deepen their studies to identify storage media for avulsed teeth that are as natural and accessible to the population as possible.

The general public, who has wide access to information, both through the media and through the internet, must know that the dented tooth can be replanted, and for this purpose, it must be stored in an appropriate environment until the time it is presented to the doctor. The consequences that dental avulsion generates motivate researchers to identify solutions that improve the oral health of affected patients.

## Figures and Tables

**Table 1:** The chemical products, reagents for AA%, and the equipment used to obtain the extracts from *M. alba L*. fruits.

Chemicals	– Aluminum chloride, – Sodium acetate, – Folin–Ciocalteu phenol reagent, – Ethanol, – Methanol, – Rutin, and – Gallic acid, were purchased from Sigma–Aldrich ultrapure water (millipore water system).

Reagents for antioxidant activity/radical scavenging activity determination	– Luminol (5-amino-2,3-dihydro-1,4-phthalazinedionine-H_2_O_2_ in buffer TRIS-HCI, at pH 8,6 for chemiluminescence tehnique (CL).

Equipment	– Sohlet system for crude extracts, – Spectrophotometer UV-Vis, Jasco V-570, for quantitative determination of flavonoids and total polyphenols, – Digital Rotary Evaporator RE100-Pro LCD (Dragon Laboratory Instruments Limited), – Chemiluminometer (Sirius Luminometer Berhelot–Gmbh Germany) for antioxidant activity measurements by CL.

**Table 2:** Determination of flavonoids and total polyphenols from fruit of white mulberry.

Popular name	Scientific name	Test code	Determination of flavonoids (in rutin expressed) (%)	Determination of total polyphenols (in gallic acid) (%)	AA (%)
Fruit of white mulberry	*M. alba* L.	E1	0.2517 2.537 μg/g	0.4271 4.271 μg/g	77.40

Fruit of white mulberry	*M. alba* L.	E2	0.9876 9.876 μg/g	1.2739 12.739 μg/g	84.79

**Table 3:** Concentrations tested for the extract E1 and extract E2.

Extract	Test code C 50 μg/mL	Test code C 150 μg/mL	Test code C 250 μg/mL
Extract E1	E1A	E1B	E1C
Extract E2	E2A	E2B	E2C

**Table 4:** Multiple comparisons post hoc LSD.

Dependent	Variable	Mean difference	Std. error	Sig.	Interval lower	Interval upper
E1 LSD	E1A E1B	−0.2447000	0.1197657	0.071	−0.464244	−0.025156
	E1A E1C	−0.0124833	0.1197657	0.919	−0.232027	−0.20
	E1B E1C	−0.2322167	0.1197657	0.084	0.012673	0.451761

E2 LSD	E2A E2B	0.3902751	0.1693948	0.047	−0.079755	−0.700795
	E2A E2C	−0.268749	0.1693948	0.047	−0.579195	−0.041845
	E2B E2C	−0.6589500	0.1693948	0.004	−0.969470	0.348430

*Note:* The mean difference is significant at the 0.1 level.

## Data Availability

The data that support the findings of this study are available from the corresponding author upon reasonable request.

## References

[1] (2019). *Textbook and Color Atlas of Traumatic Injuries to the Teeth*.

[2] Lam R. (2016). Epidemiology and Outcomes of Traumatic Dental Injuries: A Review of the Literature. *Australian Dental Journal*.

[3] Fouad A. F., Abbott P. V., Tsilingaridis G. (2020). International Association of Dental Traumatology Guidelines for the Management of Traumatic Dental Injuries: 2 Avulsion of Permanent Teeth.. *Dental Traumatology*.

[4] De Brier N., Vera Borra D. O., Singletary E. M., Zideman D. A., De Buck E., International Liaison Committee on Resuscitation First Aid Task Force (2020). Storage of an Avulsed Tooth Prior to Replantation: A Systematic Review and Meta-Analysis. *Dental Traumatology*.

[5] Siddiqui F. (2014). Storage Media for An Avulsed Tooth: Nature to the Rescue. *British Journal of Medical and Health Research*.

[6] Resende K. K. M., Faria G. P., Longo D. L., Martins L. J. O., Costa C. R. R. (2020). In Vitro Evaluation of Plants as Storage Media for Avulsed Teeth: A Systematic Review. *Dental Traumatology*.

[7] Poornima P., Kotari S., Sasalawad S. S., Nagaveni N. B., Roshan N. M., Reddy V. V. Subba (2015). Save Cells before Tooth Replantation: A Review. *International Journal of Contemporary Dental and Medical Reviews*.

[8] Zhang N., Cheng Y., Li F., Kang Q. (2021). Network Meta-Analysis of 10 Storage Mediums for Preserving Avulsed Teeth. *Frontiers in Medicine*.

[9] Udoye C. I., Jafarzadeh H., Abbott P. V. (2012). Transport Media for Avulsed Teeth: A Review. *Australian Endodontic Journal*.

[10] Bastos H. I. G., Vieira T. O., Melo B. T. A. (2015). Study of Biologycal Effects of Different Solutions Used as Preservatives in Dental Avulsion: An Analysis In Vitro. *European Journal of Biology and Medical Science Research*.

[11] Ercisli S., Orhan E. (2007). Chemical Composition of White (Morus Alba), Red (Morus Rubra) and Black (Morus Nigra) Mulberry Fruits. *Food Chemistry*.

[12] Gundogdu F. Muradoglua, Sensoy R. I. Gazioglu, Yilmaz H. (2011). Determination of Fruit Chemical Properties of *Morus nigra* L., *Morus alba* L. and *Morus rubra* L. by HPLC. *Scientia Horticulturae*.

[13] Rigane G., Salem R. B., Sayadi S., Bouaziz M. (2011). Phenolic Composition, Isolation, and Structure of a New Deoxyloganic Acid Derivative From Dhokar and Gemri-Dhokar Olive Cultivars. *Journal of Food Science*.

[14] Quettier-Deleu C., Gressier B., Vasseur J. (2000). Phenolic Compounds and Antioxidant Activities of Buckwheat (Fagopyrum Esculentum Moench) Hulls and Flour. *Journal of Ethnopharmacology*.

[15] Zhang H., Ma Z. F., Luo X., Li X. (2018). Effects of Mulberry Fruit (*Morus alba* L.) Consumption on Health Outcomes: A Mini-Review. *Antioxidants (Basel)*.

[16] D’Urso G., Mes J. J., Montoro P., Hall R. D., de Vos R. C. H. (2020). Identification of Bioactive Phytochemicals in Mulberries. *Metabolites*.

[17] Memon A., Memon N., Luthria D., Bhanger M., Pitafi A. (2010). Phenolic Acids Profiling and Antioxidant Potential of Mulberry (Morus Laevigata W., *Morus nigra* L., *Morus alba* L.) Leaves and Fruits Grown in Pakistan. *Polish Journal of Food and Nutrition Sciences*.

[18] Trope M. (2011). Avulsion of Permanent Teeth: Theory to Practice. *Dental Traumatology*.

[19] Gungor N., Sengul M. (2008). Antioxidant Activity, Total Phenolic Content and Selected Physicochemical Properties of White Mulberry (*Morus alba* L) Fruits. *International Journal of Food Properties*.

[20] Calín-Sánchez Á., Martínez-Nicolás J. J., Munera-Picazo S., Carbonell-Barrachina Á. A., Legua P., Hernández F. (2013). Bioactive Compounds and SensoryQuality of Black and White Mulberries Grown in Spain. *Plant Foods for Human Nutrition*.

[21] Isabelle M., Lee B. L., Ong C. N., Liu X., Huang D. (2008). Peroxyl Radical Scavenging Capacity, Polyphenolics, and Lipophilic Antioxidant Profiles of Mulberry Fruits Cultivated in Southern China. *Journal of Agricultural and Food Chemistry*.

[22] Yang X., Yang L., Zheng H. (2010). Hypolipidemic and Antioxidant Effects of Mulberry (*Morus alba* L.) Fruit in Hyperlipidaemia Rats. *Food and Chemical Toxicology*.

[23] Fagundes N. C. F., Bittencourt L. O., Magno M. B., Marques M. M., Maia L. C., Lima R. R. (2018). Efficacy of Hank’s Balanced Salt Solution Compared to Other Solutions in the Preservation of the Periodontal Ligament A Systematic Review and Meta-Analysis. *PLOS ONE*.

[24] Lim T. K. (2012). *Edible Medicinal and Non-Medicinal Plants*.

[25] Hwang J. Y., Choi S. C., Park J. H., Kang S. W. (2011). The Use of Green Tea Extract as a Storage Medium for the Avulsed Tooth. *Journal of Endodontics*.

[26] Ozan F., Tepe B., Polat Z. A., Er K. (2008). Evaluation of In Vitro Effect of *Morus rubra* (Red Mulberry) on Survival of Periodontal Ligament Cells. *Oral Surgery, Oral Medicine, Oral Pathology, Oral Radiology, and Endodontology*.

